# The role of academic factors on the development of mental illness stigma

**DOI:** 10.1192/j.eurpsy.2022.873

**Published:** 2022-09-01

**Authors:** L. Brahmi, B. Amemou, A. Adouni, A. Mhalla, L. Gaha

**Affiliations:** Fattouma Bourguiba University Hospital, Psychiatry Department, Monastir, Tunisia

**Keywords:** stigma, medical student, mental disorder

## Abstract

**Introduction:**

Stigma and discrimination can disrupt the lives of individuals with a mental illness, preventing their opportunities to become productive citizens. These Individuals must also face either an avoidant attitude by healthcare professionals or prejudices about their adherence to medications and the psychological nature of their physical symptoms.

**Objectives:**

Assess stigma in terms of explicit and implicit attitudes among medical school students and junior doctors. Evaluate academic factors and interfering with these attitudes.

**Methods:**

A cross-sectional study was conducted among students from medical schools in Tunisia.

All participants were invited to complete a brief anonymous electronic survey administered on the google forms online platform.

Data were collected using self-administered questionnaires, Stigma Measurement, Mental Illness: Clinicians’ Attitudes (MICA).

**Results:**

The sample consisted of 1028 respondents. The respondents’ mean age was 24.54 years (SD=3.7). Post-clinical students scored higher than pre-clinical students in questions 2, 6, and 12 on the rating scale. A positive significant relationship was identified with specialization in psychiatry. Residents who were specialized in family medicine, emergency, and intensive care had a higher stigma level compared to other residents (Mean score>0.51). The completion of a psychiatry clerkship did not significantly reduce the level of stigma toward people with a mental illness ( p=0.8).

**Conclusions:**

A combination of medical school experiences of psychiatry’s theoretical learning and clerkship are important factors that shape students. Awareness of this will enable educators to develop locally relevant anti-stigma teaching resources throughout the psychiatry curriculum to improve students’ attitudes towards psychiatry as a discipline and mental illness in general.

**Disclosure:**

No significant relationships.

